# Non-motor outcomes and divergent plasma metabolite changes during continuous subcutaneous foslevodopa/foscarbidopa infusion in Parkinson's disease

**DOI:** 10.1016/j.prdoa.2026.100527

**Published:** 2026-10-29

**Authors:** Kota Igari, Motoki Fujimaki, Masataka Fujimoto, Naoko Kaga, Hikari Taka, Yoshiki Miura, Takashi Hosaka, Ayako Shioya, Makoto Terada, Shinji Saiki

**Affiliations:** a Department of Neurology, Institute of Medicine, University of Tsukuba, 1-1-1 Tennodai, Tsukuba, Ibaraki 305-8575, Japan; b Jihatsu Research Unit, Tsukuba Institute for Advanced Research (TIAR), University of Tsukuba, 1-1-1 Tennodai, Tsukuba, Ibaraki 305-8577, Japan; c Center for Medical Education and Training, University of Tsukuba Hospital, 2-1-1 Amakubo, Tsukuba, Ibaraki 305-8576, Japan; d Laboratory of Proteomics and Biomolecular Science, Biomedical Research Core Facilities, Juntendo University Graduate School of Medicine, 2-1-1 Hongo, Bunkyo-ku, Tokyo 113-8421, Japan

**Keywords:** Parkinson's disease, Foslevodopa/foscarbidopa, Non-motor symptoms, Dopamine, Levodopa, Gastrointestinal symptoms

## Abstract

**Background:**

Circulating levodopa and metabolite changes during transition to continuous subcutaneous foslevodopa/foscarbidopa infusion (CSCI), and their relationships with clinical outcomes, remain incompletely characterized.

**Methods:**

In this exploratory prospective observational study, 19 patients with advanced Parkinson's disease underwent clinical assessments and fasting morning plasma sampling before CSCI and after clinically judged dose stabilization. Concentration–outcome associations were examined in exploratory post hoc analyses.

**Results:**

At a mean of 16.2 days, median Movement Disorder Society Non-Motor Rating Scale total and gastrointestinal scores decreased from 96 to 80 and 5 to 2 (adjusted *P* = 0.036 and 0.005); quality of life improved. Mean levodopa and 3-*O*-methyldopa increased 2.8-fold and 3.2-fold (both adjusted *P* < 0.001), whereas median free dopamine and 3,4-dihydroxyphenylacetic acid decreased from 10.1 to 5.2 and 575 to 248 nM (adjusted *P* = 0.003 and 0.021). The exploratory pretreatment dopamine coefficient in the baseline-score-adjusted non-motor model was −3.06 points/nM (95% confidence interval, −6.13 to 0.01; *P* = 0.051). Larger dopamine reductions were associated with lower baseline-adjusted on-treatment gastrointestinal scores, with attenuation in the catechol-*O*-methyltransferase (COMT)-related sensitivity cohort. Heteroskedasticity-robust confidence intervals for both dopamine coefficients included zero.

**Conclusions:**

These findings describe concentration changes during transition to a CSCI-based regimen. Dopamine–clinical associations were exploratory; predictive utility and causal mediation were not established.

**Registration:** UMIN000054567, registered 4 June 2024 (retrospectively).

## Highlights

• Plasma concentrations were compared before and during CSCI in 19 patients.
• Levodopa and 3-OMD increased, while free dopamine and DOPAC decreased.
• Dopamine–clinical associations were exploratory and imprecisely estimated.
• Sampling and medication changes limit causal interpretation.

## 1 Introduction

Continuous subcutaneous infusion (CSCI) of foslevodopa/foscarbidopa reduces motor fluctuations in advanced Parkinson's disease (PD) [1]. These phosphate prodrugs are hydrolyzed by alkaline phosphatases to levodopa and carbidopa, respectively. Continuous delivery bypasses gastrointestinal absorption and maintains systemic exposure to both active compounds [2]. Peripheral levodopa can be decarboxylated to dopamine, subsequently metabolized to 3,4-dihydroxyphenylacetic acid (DOPAC), or *O*-methylated to 3-*O*-methyldopa (3-OMD) by catechol-*O*-methyltransferase (COMT). Carbidopa inhibits peripheral decarboxylation; COMT inhibitors suppress 3-OMD formation. Concentration changes during treatment transition may reflect combined effects of delivery, dose, treatment duration, and concomitant medication changes.

Stable levodopa and high 3-OMD concentrations during levodopa–carbidopa intestinal gel infusion [3], and elevated 3-OMD during CSCI [4], motivate measuring circulating metabolites alongside levodopa. Clinical improvement after adding or reintroducing opicapone during CSCI has also been described in two patients [5]. Within-patient changes in multiple metabolites, including free dopamine, remain incompletely characterized.

Non-motor outcomes during CSCI vary across domains and individuals [6], [7]. Peripheral dopamine influences gastrointestinal (GI) function, and medication-related nausea can contribute to non-motor symptom burden [8]. This provides a rationale for exploring dopamine–clinical associations without assuming that plasma concentrations reflect local enteric signaling. We prospectively characterized plasma concentration changes alongside clinical assessments. Post hoc exploratory analyses examined pretreatment dopamine in relation to subsequent non-motor symptom burden, and dopamine changes in relation to GI symptom burden.

## 2 Methods

### 2.1 Study design and treatment

This exploratory, single-center prospective observational study enrolled 19 patients with advanced PD admitted to the University of Tsukuba Hospital for CSCI initiation between March 2024 and January 2026. Eligible patients were recruited consecutively without exception. They met Movement Disorder Society diagnostic criteria and had inadequately controlled motor fluctuations or dyskinesia despite optimized oral therapy. Treatment decisions were independent of study participation. Ethics approval (R04–275), written consent, and parent-study registration (UMIN000054567) were obtained. Sample size reflected patient availability and research funding; no formal sample-size calculation was performed.

Baseline assessments followed inpatient acclimatization (5.3 ± 3.3 days). Follow-up followed specialist-judged dose stabilization: best achievable control compatible with daily activities, with no further titration considered necessary then; no numerical criterion was used. All patients met this clinical definition before sampling. CSCI was administered for 24 h/day in 18 patients and 06:00–21:00 in P08. P08 was sampled at 09:00 after restarting at 06:00, before morning oral medication; no levodopa was taken overnight.

Concomitant levodopa/carbidopa tablets (Menesit; levodopa:carbidopa, 10:1) were used in eight patients when necessary. COMT inhibitors were discontinued after baseline sampling and before CSCI, following Japanese prescribing information [9]. Other adjuncts were unchanged, reduced, or discontinued; none were increased (Tables S2 and S2b). Programmed CSCI-derived levodopa equivalent dose (LED) excludes extra doses. Regular hypnotic, antiemetic, and laxative prescriptions were unchanged; actual as-needed use could not be comprehensively ascertained.

### 2.2 Plasma measurements and clinical assessments

Fasting morning samples were collected at both assessments. At baseline, 17 patients were sampled after morning levodopa and one before it; timing was unavailable for one. The median interval since the last dose was 142 min (interquartile range [IQR], 103–160; *n* = 18). During CSCI, all samples preceded morning oral medication with infusion running: 17 in hospital and two after discharge. For inpatients, programmed rates remained unchanged after sampling until discharge. Baseline post-dose sampling and ongoing-infusion sampling represented different pharmacokinetic conditions.

Cooled EDTA-2Na plasma was stored in liquid nitrogen. Levodopa and six metabolites were quantified by liquid chromatography–tandem mass spectrometry (LC-MS/MS) [10]; dopamine was free, unconjugated dopamine. 3,4-Dihydroxyphenylacetaldehyde (DOPAL) was below quantification throughout and excluded. One baseline 3-methoxytyramine value was below quantification and recorded as zero.

The primary clinical outcome was change in Movement Disorder Society Non-Motor Rating Scale (MDS-NMS) total score [11], reflecting symptom burden. Non-motor fluctuations were assessed separately using the MDS-NMS Non-Motor Fluctuations subscale (MDS-NMS-NMF). Domain scores, MDS-sponsored revision of the Unified Parkinson's Disease Rating Scale (MDS-UPDRS) Parts I–IV, and the 39-item Parkinson's Disease Questionnaire (PDQ-39) were also assessed. Baseline MDS-NMS used standard four-week recall; follow-up questioning focused on symptoms during CSCI, distinguished from pretreatment symptoms. The same unblinded specialist rated Part III during clinically determined best ON. The orthostatic hypotension (OH) domain measures reported symptoms, not blood pressure.

### 2.3 Statistical analysis

Paired t or Wilcoxon tests followed Shapiro–Wilk assessment of paired differences. Benjamini–Hochberg adjustment covered six analytes, seven clinical measures, and 13 MDS-NMS domains separately. Available pairs were analyzed without imputation. Nominal significance denotes unadjusted *P* < 0.05. Item-level changes were descriptive, without significance testing. Post hoc levodopa/dopamine comparisons restricted baseline sampling to 0.5–2 h after dosing.

All concentration–symptom analyses were post hoc and hypothesis-generating. Regression modeled on-treatment scores using the corresponding baseline score and baseline concentration or concentration change (during minus before). Baseline COMT use was added to the pretreatment dopamine–MDS-NMS model as a sensitivity check. Conventional 95% confidence intervals (CIs), unadjusted *P* values, and heteroskedasticity-robust (HC3) intervals are reported.

COMT-related sensitivity analyses combined eight opicapone non-users with six patients sampled again before CSCI, 84 h after withdrawal (*n* = 14), replacing only those six baseline concentrations; further analysis excluded three entacapone users (*n* = 11). Complete COMT recovery was not assumed. Additional post hoc analyses excluded concomitant oral levodopa users (*n* = 11; Table S12) or P08 (*n* = 18; paired comparisons and two dopamine models). Detailed methods, model checks, and AI assistance are described in the Supplementary material.

## 3 Results

### 3.1 Participants and treatment transition

All 19 patients completed paired assessments (age, 63.8 ± 8.8 years; disease duration, 13.6 ± 6.0 years; Table S1), 16.2 ± 11.2 days after initiation (range, 4–43). Among the eight oral levodopa users during CSCI, doses were 50–300 mg/day (median, 100). Opicapone was stopped in 11 patients and entacapone in three (Tables S2 and S2b). Total LED did not change significantly; levodopa-derived LED increased from 1004 ± 325 to 1338 ± 470 mg/day, while median other-drug LED decreased from 480 to 270 mg/day (both nominal *P* < 0.001; Table S3). One cellulitis episode resolved with oral antibiotics.

### 3.2 Plasma concentration changes

Mean levodopa increased from 5445 to 15,353 nM and 3-OMD from 20,746 to 65,912 nM (both adjusted *P* < 0.001). Median free dopamine decreased from 10.1 to 5.2 nM and DOPAC from 575 to 248 nM (adjusted *P* = 0.003 and 0.021; Fig. 1; Tables S4–S5). 3-Methoxytyramine and homovanillic acid did not change significantly.

**Fig. 1:**
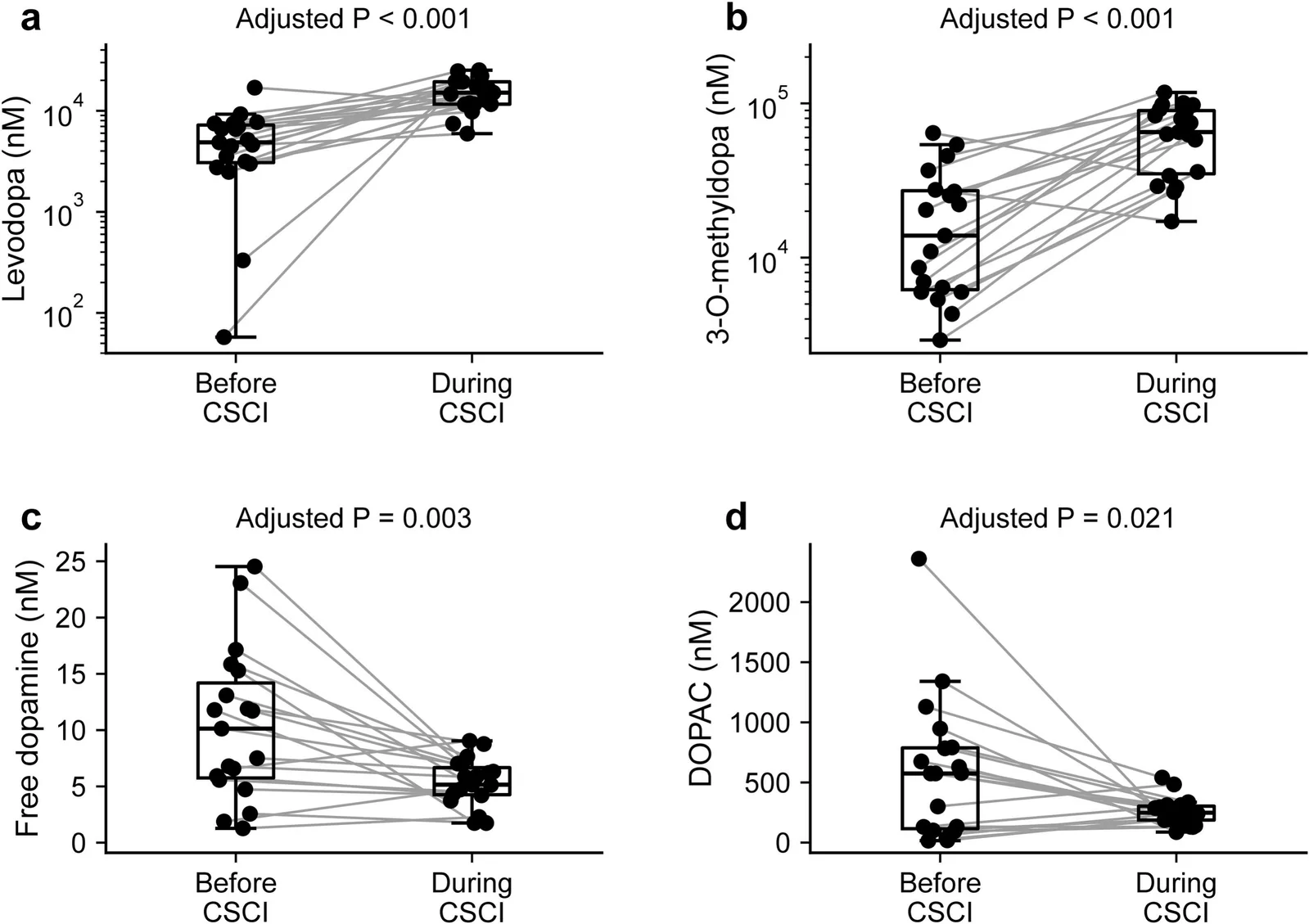
Plasma levodopa (a), 3-*O*-methyldopa (b), free dopamine (c), and DOPAC (d) before and during CSCI (*n* = 19). Panels a and b use logarithmic concentration axes. Lines connect paired observations; horizontal offsets separate overlapping points and are identical within each pair. Boxes show the interquartile range (IQR), horizontal lines the median, and whiskers the most extreme observations within 1.5 × IQR. All observations, including values beyond the whiskers, are plotted. *P* values are Benjamini–Hochberg-adjusted across the six quantifiable analytes. CSCI, continuous subcutaneous infusion of foslevodopa/foscarbidopa; DOPAC, 3,4-dihydroxyphenylacetic acid. AI-assisted computational figure preparation was reviewed and verified by the authors.

Levodopa/3-OMD increases and dopamine lowering remained nominally significant with Methods-selected tests in COMT-related sensitivity cohorts (Table S6). With baseline sampling restricted to 0.5–2 h (*n* = 6), levodopa increased (median paired ratio, 3.22; *P* = 0.015) and dopamine decreased in all six patients (median, 8.46 to 4.86 nM; *P* = 0.031). Without concomitant oral levodopa, levodopa/3-OMD increased and dopamine/DOPAC decreased; the latter two met the adjusted significance threshold with Methods-selected tests but not Wilcoxon tests (Table S12). On-treatment levodopa correlated with programmed CSCI-derived LED (ρ = 0.80; *P* < 0.001).

### 3.3 Clinical changes

Median MDS-NMS total, GI, and PDQ-39 scores decreased from 96 to 80, 5 to 2, and 30.5 to 22.4 (adjusted *P* = 0.036, 0.005, and 0.049; Fig. 2). MDS-NMS-NMF did not change significantly (adjusted *P* = 0.362; Table S3). Nausea, constipation, and drooling scores decreased in 10, 11, and eight patients; swallowing item scores increased in seven and decreased in three (Table S8). Sleep and OH improved nominally (adjusted *P* = 0.082 each); urinary and pain changes were nonsignificant (Table S7). MDS-UPDRS Parts II/IV improved nominally; best-ON Part III and OFF time did not change significantly.

**Fig. 2:**
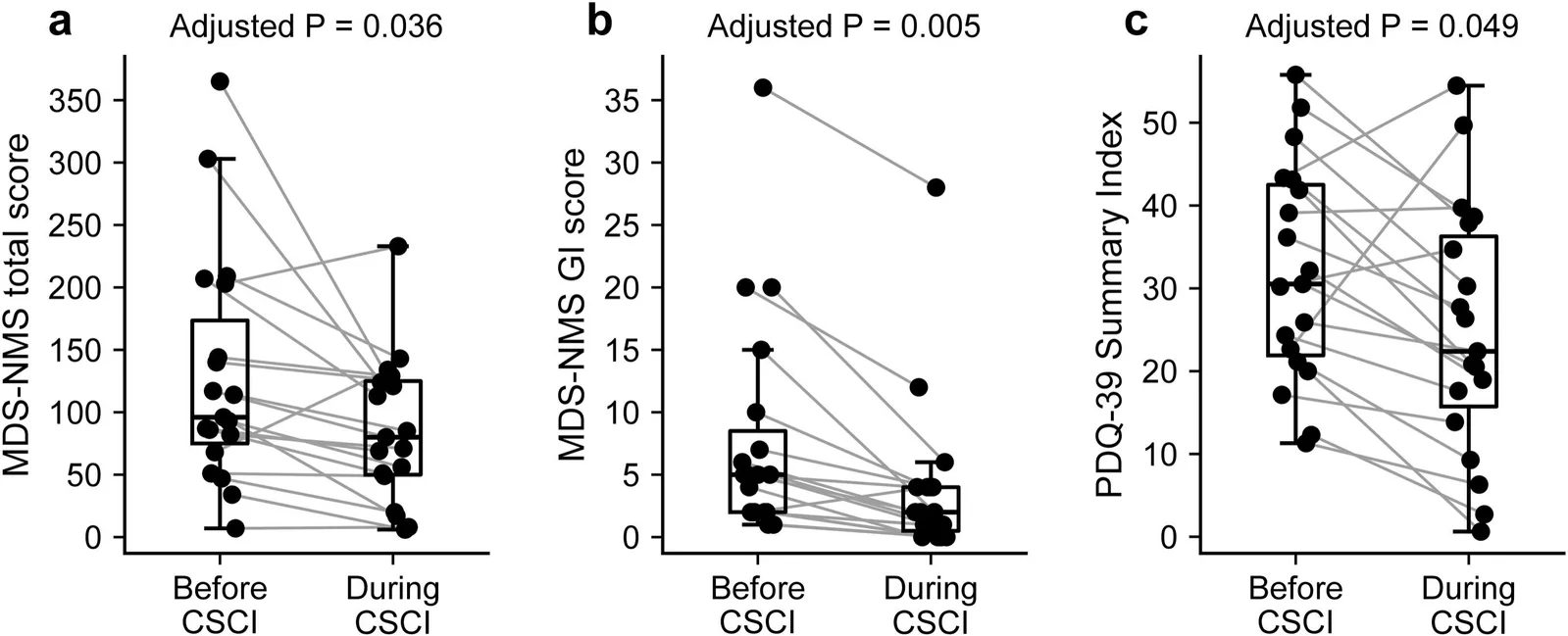
MDS-NMS total score (a), gastrointestinal domain score (b), and PDQ-39 Summary Index (c) before and during CSCI (*n* = 19). Lines connect paired observations, and horizontal offsets separate overlapping points. Boxes, medians, and whiskers are defined as in Fig. 1. All observations are plotted. *P* values are Benjamini–Hochberg-adjusted within the seven clinical measures (a, c) or the 13 MDS-NMS domains (b). CSCI, continuous subcutaneous infusion of foslevodopa/foscarbidopa; MDS-NMS, Movement Disorder Society Non-Motor Rating Scale; PDQ-39, 39-item Parkinson's Disease Questionnaire. AI-assisted computational figure preparation was reviewed and verified by the authors.

### 3.4 Exploratory post hoc associations

The baseline-score-adjusted pretreatment dopamine coefficient for on-treatment MDS-NMS total score was −3.06 points/nM (95% CI, −6.13 to 0.01; *P* = 0.051; Fig. S1). Conventional and HC3 intervals included zero; HC3 intervals also included zero across model checks (Table S9).

Larger dopamine reductions were associated with lower baseline-adjusted on-treatment GI scores (β = 0.248 points/nM; 95% CI, 0.062–0.435; *P* = 0.012), with a similar OH association. Estimates attenuated in the COMT-related sensitivity cohort, and HC3 intervals included zero (Table S10). Dopamine change correlated strongly with baseline dopamine (ρ = −0.94). Levodopa and 3-OMD showed no consistent associations with motor fluctuations or GI/OH outcomes (Tables S10–S11).

Excluding P08, four principal concentration changes remained significant after adjustment, as did MDS-NMS total and GI changes; PDQ-39 was nominally significant only. Dopamine coefficients were similar (−3.45 and 0.276 points/nM), with HC3 intervals including zero.

## 4 Discussion

Transition to a CSCI-based regimen was accompanied by increased levodopa/3-OMD and decreased free dopamine/DOPAC concentrations. Clinical assessments showed lower MDS-NMS total, GI, and PDQ-39 scores. The exploratory post hoc pretreatment dopamine coefficient was imprecisely estimated, with a conventional CI including zero. The dopamine-change–GI association was also exploratory. These findings neither establish a predictive biomarker nor demonstrate causal mediation.

Higher levodopa dosing, continuous delivery, bypassing gastrointestinal absorption, and sustained carbidopa exposure may all contribute to the higher plasma levodopa concentrations observed during CSCI, but their respective contributions cannot be disentangled in this study. Levodopa-derived LED increased approximately 33%, while plasma levodopa increased 2.8-fold. Sampling-time and treatment effects cannot be separated. COMT withdrawal may contribute to higher 3-OMD, without its contribution being quantified by sensitivity analyses. Sustained carbidopa exposure [2] offers one possible explanation for lower plasma dopamine concentrations, indirectly supported by observations with higher oral carbidopa-to-levodopa ratios [12]. Carbidopa was not measured. The decreases in plasma free dopamine and DOPAC concern peripheral circulating concentrations and do not, by themselves, establish reduced peripheral dopamine formation.

Non-motor findings broadly accord with previous cohorts [6], [7], but lower total scores do not demonstrate improved non-motor fluctuations. GI item changes were descriptive and swallowing changes mixed. The exploratory dopamine–GI association attenuated in the COMT-related sensitivity cohort, with HC3 intervals including zero. Dopamine's involvement in medication-related nausea [8] provides biological context; the regression assessed a composite GI score, not nausea specifically. GI symptoms are multifactorial, plasma dopamine may not reflect enteric signaling, and objective gastric motility was not assessed. Dopamine reduction and GI improvement may be parallel consequences of treatment transition.

This small, single-center, uncontrolled, unblinded study limits power, precision, and robustness, particularly for the post hoc dopamine analyses. Nonsignificant findings do not establish absence of change or association. Pretreatment dopamine may reflect medication exposure as well as peripheral metabolism. Multiple comparisons and the strong correlation between dopamine change and baseline dopamine further limit interpretation.

Follow-up was heterogeneous (4–43 days); clinical stabilization did not establish a concentration or response plateau. Timing may influence concentrations and symptoms, including quality of life. Different sampling positions on concentration–time profiles may partly explain concentration differences. Restriction to 0.5–2 h neither standardized oral doses nor established individual peaks, and did not eliminate this limitation. Posture and rest were incompletely standardized. Single samples cannot characterize daily exposure, distinguish formation from clearance, or assess tissue dopamine activity.

COMT withdrawal and other medication changes, including MAO-B inhibitors and zonisamide, prevent isolation of individual drug contributions or an independent CSCI effect. Adjunctive COMT inhibition, reported elsewhere [4], [5], was not evaluated. We could not distinguish improvement in PD-related manifestations from relief of medication-related adverse effects. Unmeasured activity and differences in medication administration between inpatient and outpatient settings may influence symptoms and concentrations. Daily routines, diet, sleep, bowel care, expectations, and regression to the mean may also affect symptoms. Incompletely ascertained as-needed medication use may contribute to sleep/GI changes.

Follow-up MDS-NMS used a nonstandard, variable recall period focused on symptoms during CSCI. Its measurement properties were not evaluated; differences in recall and frequency ratings may affect baseline comparability and estimated changes.

This prospective observational study documents increased plasma levodopa and 3-OMD and decreased free dopamine and DOPAC during transition to a CSCI-based regimen. Clinical assessments also showed reduced non-motor symptom burden. The dopamine–clinical associations remain exploratory and require confirmation in independent cohorts with prespecified analyses; predictive utility and causal mediation were not established.

## Ethics approval and consent

This study was approved by the Clinical Research Ethics Review Committee of the University of Tsukuba Hospital (approval number R04–275; initial approval 19 April 2023, latest amendment 13 March 2026) and was conducted in accordance with the Declaration of Helsinki. The parent observational study was registered with the University Hospital Medical Information Network Clinical Trials Registry (UMIN000054567; registered 4 June 2024, retrospectively). All participants provided written informed consent before enrollment. The manuscript contains no identifiable patient data, images, or videos.

## Declaration of generative AI and AI-assisted technologies in the manuscript preparation process

During the preparation of this work the authors used Claude (Anthropic; June 2026) and ChatGPT (OpenAI; September–October 2026) in order to assist with language editing, restructuring, translation, formatting, checking against submission requirements, figure preparation, and statistical analysis and verification. After using these tools, the authors reviewed and edited the content as needed and take full responsibility for the content of the published article.

## CRediT authorship contribution statement

**Kota Igari:** Writing – original draft, Investigation, Formal analysis, Conceptualization, Methodology, Validation, Visualization, Writing – review & editing. **Motoki Fujimaki:** Writing – review & editing, Supervision, Investigation, Conceptualization, Methodology, Validation, Project administration. **Masataka Fujimoto:** Investigation, Writing – review & editing. **Naoko Kaga:** Methodology, Investigation, Writing – review & editing. **Hikari Taka:** Methodology, Investigation, Writing – review & editing. **Yoshiki Miura:** Methodology, Investigation, Writing – review & editing. **Takashi Hosaka:** Investigation, Writing – review & editing. **Ayako Shioya:** Investigation, Writing – review & editing. **Makoto Terada:** Investigation, Writing – review & editing. **Shinji Saiki:** Writing – review & editing, Supervision, Funding acquisition, Conceptualization.

## Funding

This work was supported by the 10.13039/501100004330Smoking Research Foundation; the 10.13039/501100001691Japan Society for the Promotion of Science (JSPS) KAKENHI [grant numbers 23K17454 and 25K24671]; and the 10.13039/100009619Japan Agency for Medical Research and Development (AMED) [grant numbers JP26wm0625503 and JP25zf0127005]. The funders had no role in study design, data collection, analysis or interpretation, writing of the report, or the decision to submit the article for publication.

## Declaration of competing interest

Shinji Saiki has received research funding from the Smoking Research Foundation, JSPS, and AMED, which also supported the present work (see Funding). Motoki Fujimaki is a co-investigator in AMED ToRA-SEED (grant number JP256f0137008), which supports research time and the departmental research environment. The other authors declare that they have no known competing financial interests or personal relationships that could have appeared to influence the work reported in this paper.

## Data Availability

De-identified individual-level data underlying the reported results, including figure source data, are provided in Supplementary Data 1. Statistical methods, supporting results, and source-data mapping are provided in the Supplementary material. Analysis code and additional analytical details are available from the corresponding author upon reasonable request, subject to applicable institutional requirements.

## Appendix A Supplementary data

Supplementary material 1 De-identified participant-level clinical, medication, and plasma metabolite data; questionnaire item scores; figure source data and model parameters; and a data dictionary.

Supplementary material 2 Supplementary methods, supplementary references, Tables S1-S12, including S2b, Fig.S1 (exploratory post hoc relationship between pretreatment free dopamine and non-motor response), and source-data mapping.
