# Supplementary material for: Non-motor outcomes and divergent plasma metabolite changes during continuous subcutaneous foslevodopa/foscarbidopa infusion in Parkinson's disease
Source: Clin Park Relat Disord. 2026 Oct 29;15:100527. doi: 10.1016/j.prdoa.2026.100527 (PMC13756515; doi:10.1016/j.prdoa.2026.100527)
Supplement: Supplementary file 1 [file mmc2.docx]

**Supplementary material**

Non-motor outcomes and divergent plasma metabolite changes during continuous subcutaneous foslevodopa/foscarbidopa infusion in Parkinson’s disease

Clinical Parkinsonism & Related Disorders — Short Communication

Kota Igari, Motoki Fujimaki, Masataka Fujimoto, Naoko Kaga, Hikari Taka, Yoshiki Miura, Takashi Hosaka, Ayako Shioya, Makoto Terada, Shinji Saiki

Corresponding author: Motoki Fujimaki, MD, PhD, Department of Neurology, Institute of Medicine, University of Tsukuba, 1-1-1 Tennodai, Tsukuba, Ibaraki 305-8575, Japan. Email: mtfujima@md.tsukuba.ac.jp

Contents. Supplementary methods; supplementary references; Tables S1–S12, including S2b; Fig. S1; source-data mapping. De-identified individual-level data and source data for all figures are supplied in Supplementary Data 1 (xlsx).

**Table of contents**

Supplementary methods

Supplementary references

Table S1. Participant characteristics and CSCI treatment parameters

Table S2. Changes in antiparkinsonian medications other than CSCI

Table S2b. Patient-level medication changes

Table S3. Clinical measures and levodopa equivalent doses before and during CSCI

Table S4. Plasma levodopa and metabolite concentrations before and during CSCI

Table S5. Within-patient plasma concentration changes

Table S6. Concentration sensitivity analyses

Table S7. MDS-NMS domain scores before and during CSCI

Table S8. Changes in gastrointestinal and orthostatic symptom items

Table S9. Post hoc baseline-analyte associations with subsequent clinical response

Table S10. Concentration changes and GI/OH outcomes

Table S11. Exploratory associations with motor fluctuations

Table S12. Concentrations without concomitant oral levodopa

Fig. S1. Baseline free dopamine and change in MDS-NMS total score

Source-data mapping

**Supplementary methods**

**Study procedures and clinical assessments**

This section supplements the Methods of the main text. Patients met Movement Disorder Society clinical diagnostic criteria for Parkinson’s disease (PD) [S5], had inadequately controlled motor fluctuations or dyskinesia despite optimized oral therapy, and were considered suitable for CSCI. Eligible patients were recruited consecutively without exception. Sample size reflected practical feasibility, including patient availability and research funding; no formal sample-size calculation was performed. Baseline assessments followed inpatient acclimatization (5.3 ± 3.3 days after admission). Follow-up assessments and blood sampling followed specialist-judged dose stabilization: best achievable symptom control compatible with daily activities, with further titration not considered necessary at that time, without a numerical criterion. All patients met this clinical definition. Seventeen were assessed in hospital and two after discharge (P03 and P11). For inpatients, programmed infusion rates remained unchanged after sampling until discharge. Time from initiation to assessment was 16.2 ± 11.2 days (range, 4–43); this is not the duration spent at an unchanged dose. Sex was recorded as biological sex from medical records; sex-stratified analyses were not performed because of the small sample. Pre-enrollment screening counts were not available.

Part III of the Movement Disorder Society-sponsored revision of the Unified Parkinson’s Disease Rating Scale (MDS-UPDRS) was assessed by the same movement disorders specialist, who was not blinded to treatment status. The best-ON state was determined clinically rather than by a fixed interval after medication administration. Baseline assessments were performed after the usual oral medication dose, and assessments during treatment were performed under stable continuous infusion. The 39-item Parkinson’s Disease Questionnaire (PDQ-39) Summary Index was calculated as the mean of the eight domain scores, each expressed as a percentage of its maximum possible score (0–100). The Mini-Mental State Examination (MMSE) and Frontal Assessment Battery (FAB) were also administered (Table S1). Additional outcome measures included MDS-UPDRS Part I and the Non-Motor Fluctuations subscale (MDS-NMS-NMF) of the Movement Disorder Society Non-Motor Rating Scale (MDS-NMS). The primary clinical outcome was change in MDS-NMS total score, reflecting overall non-motor symptom burden; MDS-NMS-NMF assessed fluctuations separately. Baseline MDS-NMS assessments used the standard four-week recall. Follow-up questioning focused on symptoms during CSCI, with patients prompted to distinguish these from pretreatment symptoms. This nonstandard, variable recall period was not evaluated for measurement properties.

Foslevodopa/foscarbidopa was administered as a continuous subcutaneous infusion for 24 hours/day in 18 patients and 15 hours/day in one (P08, 06:00–21:00 at 0.2 mL/h; nighttime rate 0 mL/h). The initial CSCI dose was determined according to the prescribing information [S1]. Infusion rates and extra doses were adjusted according to motor fluctuations, OFF symptoms, dyskinesia, and tolerability. Oral levodopa/carbidopa tablets (Menesit; levodopa:carbidopa ratio, 10:1) were used in eight patients when clinically necessary. P08 usually took 100 mg levodopa at 08:00, 12:00, and 18:00; no levodopa was taken during the non-infusion period. Other antiparkinsonian medications were continued, reduced, or discontinued at the treating physician’s discretion; none were increased (Tables S2 and S2b). Levodopa equivalent doses (LEDs) were calculated using published conversion factors [S7] and the prescribing information [S1]. The CSCI-derived LED was calculated as [(daytime infusion rate in mL/h × 15 h) + (nighttime infusion rate in mL/h × 9 h)] × 170 mg/mL. This value represents the programmed continuous infusion and does not include extra doses.

Regular prescriptions for hypnotics, antiemetics, and laxatives were unchanged. Actual use of as-needed medications could not be comprehensively ascertained.

**Blood sampling**

Water was permitted during fasting. Before baseline sampling, oral medication intake was restricted to levodopa when clinically necessary. Recorded baseline sampling times ranged from 07:40 to 10:50. Recorded dosing times indicated that 17 patients were sampled after a morning oral levodopa dose. Patient P05 was sampled before the morning dose, 14 hours and 15 minutes after the recorded last dose on the previous evening. The median recorded interval from the last oral levodopa dose to baseline sampling was 142 minutes (interquartile range [IQR], 103–160 minutes; records available in 18 patients). Among the 17 sampled after morning levodopa, the median interval was 135 minutes (IQR, 100–160 minutes). During CSCI, all samples were collected with the infusion running and before morning oral medication. In P08, infusion restarted at 06:00 on the sampling day and blood was collected at 09:00, three hours after restart. The scheduled 08:00 oral dose was withheld until after sampling. Baseline sampling after intermittent oral treatment and sampling during ongoing infusion were not pharmacokinetically equivalent.

Individual levodopa during/before concentration ratios ranged from 0.69 to 336.24. The largest ratio occurred in P05, whose baseline levodopa concentration was 57.6 nM and whose baseline sample was collected before the morning dose.

Venous blood was collected into 7-mL EDTA-2Na tubes (SRL, Tokyo, Japan), kept at 4 °C for 30–60 minutes, and centrifuged at 2,660 × g for 10 minutes. The separated plasma was stored in liquid nitrogen until analysis.

**Opicapone sensitivity analysis**

At baseline, 11 patients were receiving opicapone and three entacapone. COMT inhibitor discontinuation is described in the main Methods. Partway through the study, an additional pre-CSCI blood sample was obtained in six patients receiving opicapone, 84 hours after the last dose, to provide baseline measurements with less residual catechol-*O*-methyltransferase (COMT) inhibition.

The 84-hour interval was a pragmatic clinical withdrawal interval allowing repeat sampling during hospitalization. Muthuraman et al. [S4] describe opicapone withdrawal for more than 24 h before levodopa challenge. Such clinical withdrawal intervals do not imply complete biochemical recovery: COMT inhibition can persist after opicapone is no longer detectable in plasma [S6]. Accordingly, the repeat measurements address sensitivity to recent opicapone use without assuming complete COMT recovery.

The sensitivity cohort combined eight patients not receiving opicapone and six with repeat samples 84 h after discontinuation (*n* = 14). Only the six baseline concentrations were replaced; clinical scores were unchanged. A further analysis excluded the three baseline entacapone users (P14, P16, and P17; *n* = 11). Table S6 distinguishes the Methods-selected primary paired test from the additional test. In the baseline dopamine–MDS-NMS analysis, the same 14 patients were also analyzed using their original samples to separate cohort composition from sample substitution (Table S9).

**Liquid chromatography–tandem mass spectrometry (LC-MS/MS) analysis**

Plasma levodopa, dopamine, 3-*O*-methyldopa, 3-methoxytyramine, 3,4-dihydroxyphenylacetaldehyde (DOPAL), 3,4-dihydroxyphenylacetic acid (DOPAC), and homovanillic acid were measured using a modification of our previously reported method [S2]. Dopamine was quantified in its free, unconjugated form without hydrolysis. Plasma (200 µL) was mixed with 600 µL of acetonitrile containing 0.1% formic acid and stable isotope-labeled internal standards. The internal standards were 3-methoxytyramine-1,1,2,2-d4 (5 pmol) and homovanillic acid-2,2,2′,5′,6′-d5 (200 pmol; Isosciences, Ambler, PA, USA); dopamine-1,1,2,2-d4 (20 pmol) and levodopa-2′,5′,6′-d3 (20 pmol; Cambridge Isotope Laboratories, Tewksbury, MA, USA); DOPAC-d5 (100 pmol; FUJIFILM Wako Pure Chemical Corporation, Osaka, Japan); and 3-*O*-methyldopa-d3 (100 pmol; SimSon Pharma, Mumbai, India). After centrifugation at 14,000 × g for 10 minutes at 4 °C, the supernatant was dried under a stream of nitrogen and stored at −80 °C. Immediately before analysis, the residue was reconstituted in 40 µL of mobile phase A.

Analyses were performed using an LCMS-8060 triple-quadrupole mass spectrometer coupled to a Nexera system (Shimadzu, Kyoto, Japan) and a Discovery HS F5-3 column (15 cm × 2.1 mm, 3 µm; Sigma-Aldrich, St. Louis, MO, USA). Mobile phase A consisted of 0.1% formic acid and 2 mM ammonium acetate in water; mobile phase B consisted of 0.1% formic acid and 2 mM ammonium acetate in 90% acetonitrile. The flow rate was 150 µL/min, and the column temperature was 40 °C. Mobile phase B was held at 0% for three minutes, increased to 90% at three percentage points per minute, and held at 90% for 15 minutes. Analytes were quantified using multiple reaction monitoring.

DOPAL was below the lower limit of quantification in all samples and was excluded from statistical analyses. The other metabolites were quantified using analyte-specific calibration curves. The lower limit of quantification was defined by a signal-to-noise ratio of at least 10. Values below this limit were recorded and analyzed as zero. Apart from DOPAL, the only value below the lower limit of quantification was baseline 3-methoxytyramine in one patient (P02); no other analyte had values below this limit in the original or repeat samples.

**Statistical analysis**

Paired differences were assessed using Shapiro–Wilk tests. Paired *t* tests were used when *P* > 0.05 and Wilcoxon signed-rank tests otherwise. Wilcoxon tests were exact when differences contained neither zeros nor tied absolute values; otherwise a tie-corrected normal approximation without continuity correction was used, excluding zero differences. Clinical-score differences were rounded to six decimal places before ranking; the PDQ-39 comparison contained a tie. Benjamini–Hochberg adjustment was applied separately to six analytes, seven clinical measures, and 13 MDS-NMS domains. Available pairs were analyzed without imputation. Tests were two-sided. Nominal significance refers to an unadjusted *P* < 0.05.

Concentration ratios were during/before values for each patient, summarized by the median and IQR with patient-level percentile bootstrap CIs (20,000 resamples). Post hoc paired levodopa and dopamine comparisons used original baseline samples obtained 0.5–2 h after the recorded last oral dose (n = 6), an early post-dose window informed by immediate-release pharmacokinetics in healthy adults [S3]. This window did not standardize oral doses, establish individual peak concentrations, or match pharmacokinetic states. All 19 patients remained in the primary analyses; the restricted analysis omitted 10 sampled more than 2 h after dosing, two sampled less than 0.5 h after dosing, and one with unavailable timing. In these six patients (P02, P03, P04, P07, P10, and P11), dopamine decreased from a median of 8.46 nM (IQR, 6.13–14.00) to 4.86 nM (IQR, 4.37–5.68); all six values decreased, and the median paired ratio was 0.71. Paired dopamine differences were non-normal (Shapiro–Wilk P = 0.025); the Methods-selected exact two-sided Wilcoxon P was 0.031 (paired-t P = 0.073 as an alternative check). These unadjusted post hoc comparisons address concentration changes only. Clinical and item-level changes were described using paired scores and numbers improving or worsening. DOPAL was excluded as stated above.

A post hoc sensitivity analysis excluded the eight patients receiving concomitant oral levodopa during CSCI, leaving 11 patients (Table S12). Original pre-CSCI and during-CSCI samples were used; no baseline samples were replaced with the 84-h post-opicapone-withdrawal samples, and no restriction on the baseline dose-to-sampling interval was applied. The primary test for each of the six quantifiable analytes followed the paired-difference normality criterion described above. To assess sensitivity to test choice, exact Wilcoxon signed-rank tests were also applied to all six analytes. Nominal P values and Benjamini–Hochberg-adjusted P values across the six analytes are reported separately for the primary-test and all-Wilcoxon analyses. These adjustments do not control multiplicity across all post hoc analyses.

A separate post hoc sensitivity analysis excluded P08, the patient receiving 15 h/day infusion (n = 18). Original baseline samples were retained. Paired concentration and clinical comparisons used the same test-selection and multiplicity rules as the full cohort. All six analytes, seven clinical measures, and 13 MDS-NMS domains were recalculated within their original adjustment families. The two principal dopamine regressions were repeated with on-treatment MDS-NMS total or GI score as the outcome and the corresponding baseline score as a covariate; conventional and HC3 inference used residual-degree-of-freedom t distributions.

All concentration–symptom analyses were post hoc, including the focus on gastrointestinal (GI) symptoms. Spearman symptom correlations used 199,999 two-sided random permutations with a plus-one correction and 20,000 patient-level bootstrap resamples for percentile CIs; constant bootstrap samples were discarded. Changes were during minus before CSCI. Regression models used on-treatment scores as outcomes, the corresponding baseline score, one analyte predictor, and an intercept. For identical observations and covariates, this is algebraically equivalent to a baseline-adjusted change-score model; it does not independently resolve mathematical dependence, regression to the mean, or measurement error. Relative to an improvement outcome (before minus during), the analyte coefficient changes sign, while its standard error and P value are unchanged. Baseline-score coefficients and overall model fit are not interpreted as evidence of an analyte effect. Conventional and HC3 inference used t distributions with residual degrees of freedom. Dopamine coefficients are per 1 nM; levodopa and 3-OMD coefficients are per 10,000 nM where indicated. The models describe associations and were not externally validated predictors.

Baseline COMT use (opicapone or entacapone: yes/no) was added to the baseline dopamine–MDS-NMS model. All users subsequently discontinued COMT inhibition, so this indicator cannot separate pre-treatment use, withdrawal, and associated patient characteristics. Additional checks used natural-log dopamine or included baseline levodopa. The untransformed model remains the reference. Table S9 presents all six baseline-analyte comparisons and the NMS model checks. Unadjusted estimates are emphasized; within-family Benjamini–Hochberg values do not control study-wide multiplicity.

Main figures were generated in Python with Matplotlib. Paired plots show all observations with consistent horizontal offsets. In Fig. S1, displayed change equals observed MDS-NMS change minus the change-model baseline-score coefficient multiplied by the difference between individual and cohort-mean baseline score. That coefficient is the on-treatment-model baseline coefficient minus one. The line and conventional confidence band use the full baseline-adjusted model; the band describes the fitted mean, not individual predictions. The Source_Figure3 worksheet in Supplementary Data 1 supplies the plotted values, coefficients, and covariance matrix. The README worksheet provides software versions and resampling seeds. Claude (Anthropic; June 2026) and ChatGPT (OpenAI; September–October 2026) assisted with analysis and plotting code and verification. These dates identify the versions at the month level, as reported by the authors; exact model identifiers were not available. Figures were computed from the supplied participant observations, with author review of code, numerical outputs, and figures. AI tools were not used to invent observations or generate primary research images.

**Supplementary references**

[S1] AbbVie GK, VYALEV Combination Injection: electronic prescribing information [Japanese]. https://www.pmda.go.jp/PmdaSearch/iyakuDetail/112130_1169500A1023_1_02, 2024 (accessed 8 October 2026).

[S2] A. Chukyo, O. Komori, M. Fujimaki, N. Kaga, H. Taka, Y. Li, et al., A regression analytical model of serum levodopa metabolic profile in Parkinson’s disease, Heliyon 12 (2026) e44615. https://doi.org/10.1016/j.heliyon.2026.e44615.

[S3] A. Hsu, H.M. Yao, S. Gupta, N.B. Modi, Comparison of the pharmacokinetics of an oral extended-release capsule formulation of carbidopa-levodopa (IPX066) with immediate-release carbidopa-levodopa (Sinemet), sustained-release carbidopa-levodopa (Sinemet CR), and carbidopa-levodopa-entacapone (Stalevo), J. Clin. Pharmacol. 55 (2015) 995–1003. https://doi.org/10.1002/jcph.514.

[S4] M. Muthuraman, N. Koirala, D. Ciolac, B. Pintea, M. Glaser, S. Groppa, et al., Deep brain stimulation and L-DOPA therapy: concepts of action and clinical applications in Parkinson’s disease, Front. Neurol. 9 (2018) 711. https://doi.org/10.3389/fneur.2018.00711.

[S5] R.B. Postuma, D. Berg, M. Stern, W. Poewe, C.W. Olanow, W. Oertel, et al., MDS clinical diagnostic criteria for Parkinson’s disease, Mov. Disord. 30 (2015) 1591–1601. https://doi.org/10.1002/mds.26424.

[S6] J.F. Rocha, L. Almeida, A. Falcão, P.N. Palma, A.I. Loureiro, R. Pinto, et al., Opicapone: a short lived and very long acting novel catechol-*O*-methyltransferase inhibitor following multiple dose administration in healthy subjects, Br. J. Clin. Pharmacol. 76 (2013) 763–775. https://doi.org/10.1111/bcp.12081.

[S7] S. Schade, B. Mollenhauer, C. Trenkwalder, Levodopa equivalent dose conversion factors: an updated proposal including opicapone and safinamide, Mov. Disord. Clin. Pract. 7 (2020) 343–345. https://doi.org/10.1002/mdc3.12921.

**Table S1. Participant characteristics and CSCI treatment parameters**

| **Characteristic** | **Value** |
| --- | --- |
| Age (years) | 63.8 ± 8.8 |
| Sex, male / female, n (%) | 9 (47.4) / 10 (52.6) |
| Disease duration (years) | 13.6 ± 6.0 |
| Hoehn and Yahr stage 2 / 3 / 4, n (%) | 3 (15.8) / 13 (68.4) / 3 (15.8) |
| Duration of hospitalization (days) | 25.9 ± 13.7 |
| Time from CSCI initiation to assessment (days) | 16.2 ± 11.2 |
| Daytime CSCI infusion rate (mL/h) | 0.336 ± 0.122 |
| Nighttime CSCI infusion rate (mL/h) | 0.271 ± 0.128 |
| CSCI-derived LED (mg/day) | 1,272.3 ± 467.5 |
| Use of extra CSCI doses, n (%) | 9 (47.4) |
| Concomitant oral levodopa during CSCI, n (%) | 8 (42.1) |
| Oral levodopa dose among concomitant users (mg/day), median (range) | 100 (50–300) |
| MMSE score, before / during CSCI | 29.0 [26.5–30.0] / 29.0 [26.0–30.0] |
| FAB score, before / during CSCI | 15.0 ± 3.1 / 14.6 ± 2.5 |

Values are mean ± standard deviation (SD) or median [interquartile range (IQR)], unless otherwise indicated (*n* = 19). Assessments and blood sampling during CSCI were performed after discharge in P03 and P11. MMSE and FAB scores did not change significantly (*P* = 0.905 and *P* = 0.456, respectively). The CSCI-derived LED represents the programmed continuous infusion and does not include extra doses. CSCI, continuous subcutaneous infusion of foslevodopa/foscarbidopa; FAB, Frontal Assessment Battery; LED, levodopa equivalent dose; MMSE, Mini-Mental State Examination.

**Table S2. Changes in antiparkinsonian medications other than CSCI**

| **Medication class** | **Before CSCI** | **Continued unchanged** | **Reduced** | **Discontinued** |
| --- | --- | --- | --- | --- |
| Dopamine agonists | 15 (78.9) | 10 (52.6) | 4 (21.1) | 1 (5.3) |
| MAO-B inhibitors | 13 (68.4) | 8 (42.1) | 0 (0.0) | 5 (26.3) |
| COMT inhibitors | 14 (73.7) | 0 (0.0) | 0 (0.0) | 14 (73.7) |
| Droxidopa | 3 (15.8) | 3 (15.8) | 0 (0.0) | 0 (0.0) |
| Zonisamide | 7 (36.8) | 1 (5.3) | 0 (0.0) | 6 (31.6) |
| Amantadine | 5 (26.3) | 0 (0.0) | 1 (5.3) | 4 (21.1) |
| Istradefylline | 5 (26.3) | 0 (0.0) | 0 (0.0) | 5 (26.3) |

Values are n (%) of the full cohort (*n* = 19). The columns describe medication use during CSCI relative to baseline. All 14 patients receiving a COMT inhibitor (opicapone, *n* = 11; entacapone, *n* = 3) discontinued it before CSCI initiation. COMT, catechol-*O*-methyltransferase; CSCI, continuous subcutaneous infusion of foslevodopa/foscarbidopa; MAO-B, monoamine oxidase B.

**Table S2b. Patient-level changes in antiparkinsonian medications and levodopa equivalent doses during transition to CSCI**

| **Patient** | **COMT inhibitor before CSCI (mg/day)** | **COMT inhibitor during CSCI** | **Oral levodopa before CSCI (mg/day)** | **Oral levodopa during CSCI (mg/day)** | **LED from CSCI (mg/day)** | **CSCI rate, day/night (mL/h)** | **Extra doses** | **Dopamine agonist** | **MAO-B inhibitor** | **ZNS** | **AMA** | **IST** | **DOPS** | **Total LED before CSCI (mg/day)** | **Total LED during CSCI (mg/day)** |
| --- | --- | --- | --- | --- | --- | --- | --- | --- | --- | --- | --- | --- | --- | --- | --- |
| P01 | Opicapone 25 | Discontinued | 750 | 100 | 1,377 | 0.45/0.15 | Yes | – | – | – | – | – | C | 1,225 | 1,577 |
| P02 | Opicapone 25 | Discontinued | 600 | 100 | 652.8 | 0.16/0.16 | No | – | – | – | – | – | – | 1,170 | 752.8 |
| P03 | Opicapone 25 | Discontinued | 750 | 0 | 1,494.3 | 0.43/0.26 | Yes | C | D | D | D | – | – | 1,605 | 1,764.3 |
| P04 | Opicapone 25 | Discontinued | 1,200 | 0 | 2,590.8 | 0.68/0.56 | No | R | – | C | – | – | C | 2,440 | 2,960.8 |
| P05 | – | – | 950 | 0 | 1,632 | 0.4/0.4 | Yes | R | – | – | – | – | C | 1,110.1 | 1,738.7 |
| P06 | Opicapone 25 | Discontinued | 700 | 250 | 1,591.2 | 0.39/0.39 | Yes | C | C | – | – | – | – | 1,605 | 2,396.2 |
| P07 | – | – | 850 | 50 | 979.2 | 0.24/0.24 | Yes | C | C | – | – | D | – | 1,330.1 | 1,339.3 |
| P08 | – | – | 650 | 300 | 510 | 0.2/0 | Yes | C | D | D | – | – | – | 970 | 1,080 |
| P09 | Opicapone 25 | Discontinued | 600 | 0 | 1,045.5 | 0.29/0.2 | No | C | – | – | – | – | – | 1,006.7 | 1,152.2 |
| P10 | Opicapone 25 | Discontinued | 700 | 0 | 1,020 | 0.25/0.25 | No | D | D | D | – | – | – | 1,655 | 1,020 |
| P11 | Opicapone 25 | Discontinued | 750 | 0 | 1,224 | 0.3/0.3 | No | C | D | – | – | D | – | 1,625 | 1,524 |
| P12 | Opicapone 25 | Discontinued | 1,200 | 0 | 1,677.9 | 0.4/0.43 | No | R | D | D | – | D | – | 2,346.7 | 1,690.5 |
| P13 | – | – | 1,000 | 100 | 1,453.5 | 0.45/0.2 | Yes | – | – | – | D | – | – | 1,150 | 1,553.5 |
| P14 | Entacapone 300 | Discontinued | 600 | 300 | 1,672.8 | 0.41/0.41 | No | C | C | – | R | – | – | 1,278 | 2,352.8 |
| P15 | Opicapone 25 | Discontinued | 500 | 0 | 918 | 0.24/0.2 | Yes | – | C | – | – | – | – | 900 | 1,067.4 |
| P16 | Entacapone 700 | Discontinued | 700 | 0 | 1,264.8 | 0.31/0.31 | No | C | C | D | – | D | – | 1,767.2 | 1,814.8 |
| P17 | Entacapone 600 | Discontinued | 600 | 50 | 1,224 | 0.3/0.3 | Yes | C | C | – | – | D | – | 1,117.6 | 1,384 |
| P18 | – | – | 500 | 0 | 979.2 | 0.24/0.24 | No | C | C | – | D | – | – | 803.4 | 1,305 |
| P19 | Opicapone 25 | Discontinued | 650 | 0 | 867 | 0.25/0.15 | No | R | C | D | D | – | – | 1,982.5 | 1,557 |

Values are individual-patient data (n = 19). C, continued unchanged; R, dose reduced; D, discontinued; –, not used. Oral levodopa doses are shown as levodopa mg/day; “0” indicates no oral levodopa during CSCI. All COMT inhibitors were discontinued after baseline assessment and sampling, before CSCI initiation, in accordance with Japanese prescribing information. Doses of adjunctive medications other than COMT inhibitors and levodopa are not shown. CSCI-derived LED is calculated from the programmed continuous infusion and excludes on-demand extra doses. CSCI infusion rates are the stabilized daytime/nighttime pump rates; a nighttime rate of 0 indicates that the infusion was suspended overnight (15 h/day regimen). “Extra doses” indicates use of the pump’s on-demand extra-dose function. AMA, amantadine; COMT, catechol-O-methyltransferase; CSCI, continuous subcutaneous infusion of foslevodopa/foscarbidopa; DOPS, droxidopa; IST, istradefylline; LED, levodopa equivalent dose; MAO-B, monoamine oxidase B; ZNS, zonisamide.

**Table S3. Clinical measures and levodopa equivalent doses before and during CSCI**

| **Measure** | **Before CSCI** | **During CSCI** | **P** | **Adjusted P** |
| --- | --- | --- | --- | --- |
| MDS-UPDRS Part I | 13.2 ± 6.8 | 11.5 ± 6.1 | 0.209 | 0.292 |
| MDS-UPDRS Part II | 18.0 [12.5–25.0] | 13.0 [10.0–19.5] | 0.034 | 0.060 |
| MDS-UPDRS Part III | 28.3 ± 17.8 | 30.9 ± 18.5 | 0.436 | 0.436 |
| MDS-UPDRS Part IV | 11.1 ± 4.5 | 8.5 ± 3.8 | 0.022 | 0.051 |
| MDS-NMS total score | 96.0 [75.0–173.5] | 80.0 [50.0–125.0] | 0.005 | 0.036 |
| MDS-NMS-NMF | 24.2 ± 16.9 | 19.0 ± 22.0 | 0.310 | 0.362 |
| PDQ-39 Summary Index | 30.5 [21.9–42.5] | 22.4 [15.7–36.3] | 0.014 | 0.049 |
| Dyskinesia (h/day) | 4.5 ± 5.0 | 2.8 ± 3.5 | 0.160 | — |
| OFF time (h/day) | 4.2 ± 1.5 | 3.5 ± 2.9 | 0.325 | — |
| OFF time with dystonia (h/day) | 0.0 [0.0–1.6] | 0.0 [0.0–0.5] | 0.552 | — |
| Total LED (mg/day) | 1,425.6 ± 466.3 | 1,580.5 ± 535.7 | 0.164 | — |
| Levodopa-derived LED (mg/day) | 1,004.1 ± 324.8 | 1,338.1 ± 469.7 | <0.001 | — |
| LED from other antiparkinsonian medications (mg/day) | 480.0 [215.0–550.9] | 270.0 [103.4–347.9] | <0.001 | — |

Values are mean ± SD or median [IQR] (*n* = 19 unless otherwise indicated). The normality of paired differences was assessed using the Shapiro–Wilk test; paired *t* tests or Wilcoxon signed-rank tests were used accordingly. Adjusted P values were calculated using the Benjamini–Hochberg procedure across seven clinical measures (MDS-UPDRS Parts I–IV, MDS-NMS total score, MDS-NMS-NMF, and PDQ-39). Other measures were not adjusted for multiplicity (—). Dyskinesia, OFF time, and OFF time with dystonia were assessed in 18 patients. Levodopa-derived LED includes oral levodopa adjusted for COMT inhibitor use at baseline, and foslevodopa/foscarbidopa plus concomitant oral levodopa during CSCI. LED from other medications was calculated by subtracting levodopa-derived LED from the recorded total LED. COMT, catechol-*O*-methyltransferase; CSCI, continuous subcutaneous infusion of foslevodopa/foscarbidopa; LED, levodopa equivalent dose; MDS-NMS, Movement Disorder Society Non-Motor Rating Scale; MDS-NMS-NMF, MDS-NMS Non-Motor Fluctuations subscale; MDS-UPDRS, Movement Disorder Society-sponsored revision of the Unified Parkinson’s Disease Rating Scale; PDQ-39, 39-item Parkinson’s Disease Questionnaire.

**Table S4. Plasma levodopa and metabolite concentrations before and during CSCI**

| **Analyte (nM)** | **Before CSCI** | **During CSCI** | **P** | **Adjusted P** |
| --- | --- | --- | --- | --- |
| Levodopa | 5,445 ± 3,748 | 15,353 ± 5,413 | <0.001 | <0.001 |
| 3-*O*-methyldopa | 20,746 ± 18,148 | 65,912 ± 30,115 | <0.001 | <0.001 |
| Dopamine | 10.1 [5.7–14.2] | 5.2 [4.3–6.7] | 0.002 | 0.003 |
| 3-Methoxytyramine | 0.400 [0.116–1.122] | 0.810 [0.556–0.921] | 0.275 | 0.330 |
| DOPAL | BLQ | BLQ | — | — |
| DOPAC | 575 [116–788] | 248 [187–303] | 0.014 | 0.021 |
| Homovanillic acid | 794 ± 899 | 999 ± 523 | 0.406 | 0.406 |

Values are mean ± SD or median [IQR] (*n* = 19). Paired t or Wilcoxon signed-rank tests; Benjamini–Hochberg adjustment across six quantifiable analytes. Dopamine was measured in its free, unconjugated form. BLQ, below the lower limit of quantification (recorded as zero); CSCI, continuous subcutaneous infusion of foslevodopa/foscarbidopa; DOPAC, 3,4-dihydroxyphenylacetic acid; DOPAL, 3,4-dihydroxyphenylacetaldehyde.

**Table S5. Within-patient plasma concentration changes**

| **Cohort (n)** | **Analyte** | **Ratio median [IQR]** | **Ratio median 95% CI** | **Increased / unchanged / decreased** |
| --- | --- | --- | --- | --- |
| Full (19) | Levodopa | 2.97 [1.78–4.44] | 1.89 to 4.23 | 18 / 0 / 1 |
| Full (19) | 3-OMD | 4.85 [2.71–7.85] | 2.79 to 6.00 | 17 / 0 / 2 |
| Full (19) | Dopamine | 0.69 [0.40–0.85] | 0.48 to 0.84 | 3 / 0 / 16 |
| Full (19) | DOPAC | 0.48 [0.41–1.76] | 0.42 to 1.61 | 7 / 0 / 12 |
| Sensitivity (14) | Levodopa | 2.38 [1.34–4.55] | 1.33 to 4.65 | 12 / 0 / 2 |
| Sensitivity (14) | 3-OMD | 2.75 [2.27–3.41] | 2.20 to 3.48 | 12 / 0 / 2 |
| Sensitivity (14) | Dopamine | 0.66 [0.29–1.06] | 0.35 to 1.11 | 4 / 0 / 10 |
| Sensitivity (14) | DOPAC | 0.79 [0.43–3.11] | 0.42 to 2.84 | 6 / 0 / 8 |

Ratios are during/before CSCI for each individual, not ratios of cohort means or medians. CIs are percentile bootstrap intervals (20,000 patient resamples). The sensitivity cohort uses repeat 84-h pre-CSCI values where available, including DOPAC, with clinical scores unchanged. Levodopa ratios are sensitive to very low baseline measurements: P05 was sampled before the morning dose. Full-cohort mean paired increases were 9,907 nM (t-based 95% CI 6,368–13,447) for levodopa and 45,166 nM (30,540–59,792) for 3-OMD. 3-OMD, 3-*O*-methyldopa; CI, confidence interval; DOPAC, 3,4-dihydroxyphenylacetic acid; IQR, interquartile range.

**Table S6. Concentration sensitivity analyses**

The primary test follows the Methods criterion for paired differences. The additional test assesses sensitivity to test choice; all P values are unadjusted.

**Opicapone sensitivity cohort (*n* = 14)**

| **Analyte (nM)** | **Before CSCI** | **During CSCI** | **Primary test** | **Additional test** |
| --- | --- | --- | --- | --- |
| Dopamine | 13.1 ± 11.6 | 5.3 ± 2.3 | Wilcoxon  *P* = 0.049 | Paired t  *P* = 0.031 |
| Levodopa | 6,582.5 ± 5,485.2 | 14,722.4 ± 5,108.7 | Paired t  *P* = 0.003 | Wilcoxon  *P* = 0.002 |
| 3-OMD | 27,179.9 ± 17,772.6 | 63,340.9 ± 31,436.1 | Paired t  *P* < 0.001 | Wilcoxon  *P* = 0.002 |

**Further exclusion of baseline entacapone users (*n* = 11)**

| **Analyte (nM)** | **Before CSCI** | **During CSCI** | **Primary test** | **Additional test** |
| --- | --- | --- | --- | --- |
| Dopamine | 15.0 ± 12.4 | 5.7 ± 2.3 | Paired t  *P* = 0.043 | Wilcoxon  *P* = 0.102 |
| Levodopa | 7,400.3 ± 5,959.9 | 14,082.1 ± 5,227.7 | Paired t  *P* = 0.032 | Wilcoxon  *P* = 0.019 |
| 3-OMD | 24,947.7 ± 18,244.8 | 56,946.7 ± 32,380.5 | Paired t  *P* = 0.008 | Wilcoxon  *P* = 0.014 |

Values are mean ± SD for consistent descriptive presentation across tests. The *n* = 14 cohort combines eight patients not receiving opicapone and six with repeat baseline samples 84 h after its discontinuation. The *n* = 11 cohort additionally excludes entacapone users P14, P16, and P17. Paired *t* tests are primary when Shapiro–Wilk *P* > 0.05; otherwise Wilcoxon signed-rank tests are primary. Test designation was not based on which test gave a smaller P value. 3-OMD, 3-*O*-methyldopa; CSCI, continuous subcutaneous infusion of foslevodopa/foscarbidopa.

**Table S7. MDS-NMS domain scores before and during CSCI**

| **Domain** | **Before CSCI** | **During CSCI** | **P** | **Adjusted P** |
| --- | --- | --- | --- | --- |
| Depression | 10.6 ± 10.9 | 9.9 ± 9.2 | 0.665 | 0.720 |
| Anxiety | 13.1 ± 12.6 | 9.9 ± 10.1 | 0.245 | 0.398 |
| Apathy | 7.9 ± 8.5 | 5.5 ± 6.7 | 0.137 | 0.254 |
| Psychosis | 0.0 [0.0–5.0] | 0.0 [0.0–2.0] | 0.906 | 0.906 |
| Impulse control and related disorders | 0.0 [0.0–1.5] | 0.0 [0.0–1.0] | 0.137 | 0.254 |
| Cognition | 7.0 [3.5–34.5] | 7.0 [1.5–22.5] | 0.293 | 0.423 |
| Orthostatic hypotension | 2.0 [0.0–7.0] | 0.0 [0.0–3.0] | 0.025 | 0.082 |
| Urinary | 9.0 [3.0–19.5] | 6.0 [2.0–12.0] | 0.477 | 0.564 |
| Sexual | 1.0 [0.0–4.0] | 0.0 [0.0–0.5] | 0.012 | 0.078 |
| Gastrointestinal | 5.0 [2.0–8.5] | 2.0 [0.5–4.0] | <0.001 | 0.005 |
| Sleep and wakefulness | 15.5 ± 12.9 | 9.9 ± 10.3 | 0.021 | 0.082 |
| Pain | 9.2 ± 11.5 | 6.6 ± 9.0 | 0.387 | 0.503 |
| Other | 18.3 ± 17.8 | 10.7 ± 13.0 | 0.095 | 0.246 |

Values are mean ± SD or median [IQR] (*n* = 19). The normality of paired differences was assessed using the Shapiro–Wilk test; paired *t* tests or Wilcoxon signed-rank tests were used accordingly. Adjusted P values were calculated using the Benjamini–Hochberg procedure across the 13 MDS-NMS domains. CSCI, continuous subcutaneous infusion of foslevodopa/foscarbidopa; MDS-NMS, Movement Disorder Society Non-Motor Rating Scale.

**Table S8. Changes in gastrointestinal and orthostatic symptom items**

| **Item** | **Before median [IQR]** | **During median [IQR]** | **Improved / unchanged / worsened** | **Resolved / present before** |
| --- | --- | --- | --- | --- |
| Q34 Drooling | 1.0 [0.0–1.0] | 0.0 [0.0–0.0] | 8 / 11 / 0 | 8 / 11 |
| Q35 Swallowing | 0.0 [0.0–0.0] | 0.0 [0.0–1.0] | 3 / 9 / 7 | 3 / 4 |
| Q36 Nausea | 1.0 [0.0–1.0] | 0.0 [0.0–0.0] | 10 / 7 / 2 | 9 / 11 |
| Q37 Constipation | 4.0 [0.0–6.0] | 1.0 [0.0–2.0] | 11 / 7 / 1 | 2 / 11 |
| Q27 Lightheadedness/fainting | 0.0 [0.0–5.0] | 0.0 [0.0–1.0] | 8 / 8 / 3 | 5 / 8 |
| Q28 Dizziness/weakness on standing | 0.0 [0.0–3.0] | 0.0 [0.0–1.5] | 4 / 13 / 2 | 3 / 9 |

n = 19. Items are the stored MDS-NMS frequency × severity scores. Improvement/worsening means a lower/higher score; resolution means a positive baseline score becoming zero. Nausea and constipation scores decreased in many patients; swallowing item changes were mixed. These counts are descriptive and were not subjected to paired significance tests. Item numbers refer to the sequential Q numbers in Supplementary Data 1; shortened labels identify the constructs and do not reproduce the questionnaire. IQR, interquartile range; MDS-NMS, Movement Disorder Society Non-Motor Rating Scale.

**Table S9. Post hoc baseline-analyte associations with subsequent clinical response**

**Rank correlations**

| **Baseline analyte / outcome** | **n** | **ρ (95% CI)** | **P** | **Adjusted P** |
| --- | --- | --- | --- | --- |
| Dopamine / MDS-NMS total | 19 | −0.45 (−0.78 to 0.05) | 0.054 | 0.326 |
| Dopamine / GI | 19 | −0.10 (−0.62 to 0.49) | 0.689 | 0.704 |
| Dopamine / OH | 19 | −0.21 (−0.61 to 0.28) | 0.384 | 0.704 |
| 3-OMD / MDS-UPDRS III | 19 | 0.12 (−0.43 to 0.61) | 0.632 | 0.704 |
| 3-OMD / OFF time | 18 | 0.10 (−0.41 to 0.57) | 0.704 | 0.704 |
| 3-OMD / Dyskinesia time | 18 | −0.34 (−0.66 to 0.09) | 0.173 | 0.518 |

Correlations relate baseline concentration to during-minus-before symptom change; negative coefficients indicate greater improvement with a higher baseline concentration. P values are two-sided permutation values, and CIs are patient-level bootstrap intervals.

**Models adjusted for baseline outcome score**

| **Baseline analyte / outcome** | **β (95% CI)** | **P** | **Adjusted P** | **HC3 95% CI** | **HC3 P** |
| --- | --- | --- | --- | --- | --- |
| Dopamine / MDS-NMS total | −3.06 (−6.13 to 0.01) | 0.051 | 0.101 | −7.48 to 1.36 | 0.162 |
| Dopamine / GI | −0.25 (−0.43 to −0.08) | 0.007 | 0.044 | −0.63 to 0.13 | 0.177 |
| Dopamine / OH | −0.23 (−0.43 to −0.02) | 0.031 | 0.094 | −0.58 to 0.12 | 0.181 |
| 3-OMD / MDS-UPDRS III | 1.45 (−2.34 to 5.24) | 0.429 | 0.429 | −4.29 to 7.19 | 0.599 |
| 3-OMD / OFF time | 0.58 (−0.26 to 1.42) | 0.164 | 0.246 | −0.60 to 1.75 | 0.312 |
| 3-OMD / Dyskinesia time | −0.45 (−1.43 to 0.53) | 0.340 | 0.408 | −1.18 to 0.28 | 0.206 |

Outcome: the on-treatment score or hours/day; covariates: intercept, baseline outcome, and baseline analyte. β is per 1-nM dopamine or 10,000-nM 3-OMD increase. CIs in the β column are conventional. Adjusted P values are Benjamini–Hochberg values across the six comparisons within each table, not study-wide correction. The six comparisons were defined post hoc during revision. Unadjusted effect estimates are the main exploratory summaries; no prediction accuracy or treatment-specific effect was validated. GI, gastrointestinal; OH, orthostatic hypotension symptom domain.

**Baseline dopamine and MDS-NMS response: model and cohort checks**

| **Model or cohort** | **Dopamine β (95% CI)** | **P** | **HC3 95% CI** | **HC3 P** |
| --- | --- | --- | --- | --- |
| Reference, *n* = 19 | −3.06 (−6.13 to 0.01) | 0.051 | −7.48 to 1.36 | 0.162 |
| + COMT use, *n* = 19 | −2.95 (−5.21 to −0.69) | 0.014 | −6.06 to 0.17 | 0.062 |
| + Baseline levodopa, *n* = 19 | −2.88 (−7.99 to 2.24) | 0.249 | −8.77 to 3.02 | 0.315 |
| Same 14, original samples | −3.61 (−7.29 to 0.06) | 0.053 | −8.87 to 1.65 | 0.159 |
| Same 14, 84-h samples | −2.57 (−4.88 to −0.26) | 0.032 | −6.16 to 1.02 | 0.143 |
| Log dopamine, *n* = 19 | −29.78 (−53.40 to −6.15) | 0.017 | −70.92 to 11.37 | 0.145 |
| Log dopamine + COMT, *n* = 19 | −22.55 (−42.57 to −2.53) | 0.030 | −55.70 to 10.60 | 0.168 |

Outcome: on-treatment MDS-NMS total score. Every model includes the baseline total score. β is points per 1-nM higher dopamine for untransformed models and per natural-log unit for log models; these effect sizes are on different scales. All P values are unadjusted. COMT use means baseline opicapone or entacapone use (14 users, 5 non-users); all users subsequently discontinued treatment. The COMT coefficient in the untransformed model was −60.41 points (95% CI −93.86 to −26.97; HC3 CI −114.34 to −6.48), an observational estimate that does not isolate a withdrawal effect. The same 14-patient subset had similar rank correlations before and after substitution of six repeat 84-h samples (ρ = −0.622, permutation *P* = 0.020; and ρ = −0.618, *P* = 0.022). Thus, the stronger rank correlation than in all 19 patients cannot be attributed to the sample substitution. The cohorts overlap and are not independent validation. The COMT and log models were additional post hoc checks prompted by potential confounding and scale dependence. The reference model was retained. HC3 intervals include zero for all dopamine coefficients shown here. Source data are provided in Supplementary Data 1. 3-OMD, 3-*O*-methyldopa; COMT, catechol-*O*-methyltransferase; HC3, heteroskedasticity-robust inference; MDS-NMS, Movement Disorder Society Non-Motor Rating Scale.

**Table S10. Concentration changes and GI/OH outcomes**

**Dopamine change**

| **Domain and cohort** | **β (95% CI)** | **P** | **HC3 95% CI** | **HC3 P** |
| --- | --- | --- | --- | --- |
| GI, *n* = 19 | 0.248 (0.062 to 0.435) | 0.012 | −0.144 to 0.641 | 0.199 |
| OH, *n* = 19 | 0.248 (0.038 to 0.458) | 0.024 | −0.105 to 0.601 | 0.156 |
| GI, *n* = 14 | 0.096 (−0.067 to 0.259) | 0.222 | −0.304 to 0.496 | 0.607 |
| OH, *n* = 14 | 0.132 (−0.011 to 0.274) | 0.067 | −0.209 to 0.472 | 0.412 |

Outcome: on-treatment GI or OH domain score. Models adjust for the corresponding baseline score. Dopamine β is points per 1-nM increase in concentration change (during minus before). All P values are unadjusted. The *n* = 14 cohort uses repeat 84-h baseline concentrations where available and unchanged clinical scores.

**Levodopa and 3-OMD changes**

| **Analyte, domain, cohort** | **β (95% CI)** | **P** | **HC3 95% CI** | **HC3 P** |
| --- | --- | --- | --- | --- |
| Levodopa, GI, *n* = 19 | 1.512 (−0.359 to 3.384) | 0.106 | −2.854 to 5.879 | 0.473 |
| Levodopa, OH, *n* = 19 | 1.782 (−0.208 to 3.772) | 0.076 | −2.035 to 5.599 | 0.337 |
| 3-OMD, GI, *n* = 19 | −0.237 (−0.719 to 0.245) | 0.313 | −0.696 to 0.222 | 0.290 |
| 3-OMD, OH, *n* = 19 | −0.040 (−0.564 to 0.485) | 0.875 | −0.475 to 0.396 | 0.850 |
| Levodopa, GI, *n* = 14 | 2.118 (0.057 to 4.179) | 0.045 | −6.579 to 10.815 | 0.603 |
| Levodopa, OH, *n* = 14 | 1.491 (−0.683 to 3.665) | 0.159 | −3.537 to 6.518 | 0.527 |
| 3-OMD, GI, *n* = 14 | −0.234 (−0.941 to 0.474) | 0.483 | −0.687 to 0.220 | 0.281 |
| 3-OMD, OH, *n* = 14 | 0.048 (−0.626 to 0.723) | 0.877 | −0.645 to 0.742 | 0.880 |

The outcome, baseline-score adjustment and cohort definitions are the same as above. Levodopa and 3-OMD β values are points per 10,000-nM increase in concentration change. Each analyte was entered in a separate model. All P values are unadjusted. These estimates are exploratory and do not establish an analyte-specific mechanism. 3-OMD, 3-*O*-methyldopa; GI, gastrointestinal; OH, orthostatic hypotension symptom domain; HC3, heteroskedasticity-robust inference.

**Table S11. Exploratory associations with motor fluctuations**

| **Outcome** | **Comparison** | **Analyte** | **ρ (95% CI)** | **P** | **Adjusted P** |
| --- | --- | --- | --- | --- | --- |
| OFF | During | Levodopa | 0.26 (−0.24 to 0.66) | 0.302 | 0.796 |
| OFF | During | 3-OMD | 0.10 (−0.39 to 0.53) | 0.680 | 0.796 |
| OFF | Change | Levodopa | 0.09 (−0.48 to 0.54) | 0.707 | 0.796 |
| OFF | Change | 3-OMD | −0.28 (−0.70 to 0.27) | 0.260 | 0.796 |
| Dyskinesia | During | Levodopa | 0.25 (−0.21 to 0.66) | 0.324 | 0.796 |
| Dyskinesia | During | 3-OMD | 0.12 (−0.40 to 0.60) | 0.645 | 0.796 |
| Dyskinesia | Change | Levodopa | −0.16 (−0.64 to 0.38) | 0.517 | 0.796 |
| Dyskinesia | Change | 3-OMD | 0.14 (−0.39 to 0.62) | 0.588 | 0.796 |
| OFF | During | 3-OMD/levodopa | 0.01 (−0.50 to 0.44) | 0.979 | 0.979 |

*n* = 18 paired observations. During compares on-treatment concentrations with hours/day of symptoms; Change compares during-minus-before concentration and symptom changes. The 3-OMD/levodopa comparison uses the on-treatment concentration ratio. P values are permutation P values; CIs are patient-level bootstrap intervals. Adjusted P values use Benjamini–Hochberg correction across these nine comparisons. 3-OMD, 3-*O*-methyldopa.

**Table S12. Plasma concentration sensitivity analysis in patients without concomitant oral levodopa during CSCI**

Original paired samples from 11 patients

| **Analyte (nM)** | **Before CSCI** | **During CSCI** | **Primary P** | **Primary BH-adjusted P** | **Wilcoxon P** | **Wilcoxon BH-adjusted P** |
| --- | --- | --- | --- | --- | --- | --- |
| Levodopa | 6,785.0 ± 4,198.7 | 15,365.8 ± 4,497.6 | 0.002 | 0.007 | 0.007 | 0.021 |
| 3-OMD | 13,809.2 ± 11,183.8 | 67,230.6 ± 29,222.5 | <0.001 | <0.001 | <0.001 | 0.006 |
| Dopamine | 11.5 ± 7.7 | 5.5 ± 2.4 | 0.032 | 0.048 | 0.032 | 0.063 |
| DOPAC | 612.8 ± 439.1 | 267.5 ± 144.6 | 0.028 | 0.048 | 0.042 | 0.063 |
| 3-MT | 0.86 ± 1.59 | 0.76 ± 0.28 | 0.083 | 0.100 | 0.083 | 0.100 |
| HVA | 564.9 ± 646.7 | 808.1 ± 360.8 | 0.220 | 0.220 | 0.365 | 0.365 |

Values are mean ± SD for consistent descriptive presentation across tests; all six analytes had 11 complete pairs. Dopamine was measured in its free, unconjugated form.

The eight patients receiving oral levodopa during CSCI were excluded from the full cohort of 19. Original pre-CSCI and during-CSCI concentrations were used without 84-h baseline substitutions or an additional restriction on the baseline dose-to-sampling interval. This cohort is distinct from the COMT-related n = 11 sensitivity cohort in Table S6.

Primary tests followed the Methods criterion: paired t tests when the Shapiro–Wilk P value for paired differences was >0.05, and Wilcoxon signed-rank tests otherwise. Paired t tests were primary for all analytes except 3-MT. Exact Wilcoxon signed-rank tests were additionally applied to all six analytes to assess sensitivity to test choice; no paired differences were zero or tied in absolute value. All tests were two-sided.

BH adjustment was applied across six analytes separately for the primary-test and all-Wilcoxon analyses. These adjustments do not control multiplicity across all post hoc analyses. For dopamine and DOPAC, the adjusted P values were 0.048 with the primary tests and 0.063 with Wilcoxon tests.

BH, Benjamini–Hochberg; CSCI, continuous subcutaneous infusion of foslevodopa/foscarbidopa; 3-OMD, 3-O-methyldopa; DOPAC, 3,4-dihydroxyphenylacetic acid; 3-MT, 3-methoxytyramine; HVA, homovanillic acid.

**Fig. S1. Baseline free dopamine and change in MDS-NMS total score**

Fig. S1. Exploratory post hoc relationship between baseline free dopamine and change in MDS-NMS total score (n = 19). Changes are adjusted to the cohort mean baseline score using an ordinary least-squares model with on-treatment MDS-NMS total score as the outcome and baseline score and dopamine as predictors. Points represent adjusted changes; negative values indicate improvement on this adjusted scale. The line shows fitted change at the mean baseline score, and shading shows the conventional 95% confidence interval for the mean, not a prediction interval. β denotes points per 1-nM higher baseline dopamine; the P value is unadjusted. The estimate is uncertain; conventional and HC3 intervals for the dopamine coefficient include zero. HC3 intervals and sensitivity analyses are reported in Table S9. The calculation is described in the Supplementary methods, and the plotted values, coefficients, and covariance matrix are supplied in the Source_Figure3 worksheet of Supplementary Data 1. MDS-NMS, Movement Disorder Society Non-Motor Rating Scale. AI-assisted computational figure preparation was reviewed and verified by the authors.

**Source-data mapping (Supplementary Data 1, xlsx)**

| **Worksheet** | **Reported display or analysis** |
| --- | --- |
| Demographics_Medications | Tables S1–S3, including S2b; medication and LED changes, COMT status, dose-to-sampling intervals, and cohort definitions, including Table S12. |
| Clinical_Scores | Tables S3, S7, and S9–S11; Fig. 2 outcomes and the clinical scores used in Fig. S1. |
| Plasma_Metabolites | Tables S4–S6, S9–S12; Fig. 1; original baseline, repeat 84-h baseline, and during-CSCI concentrations. |
| Individual_Items | Table S8; underlying MDS-NMS, PDQ-39, and MDS-UPDRS item scores. |
| Source_Figure2 / Source_Figure1 | Source data for Fig. 1 / Fig. 2, respectively. Original worksheet names and column orders are retained. |
| Source_FigureS1 | Recorded timing and the 0.5–2 h subgroup flag (n = 6); paired levodopa inputs. Corresponding dopamine observations are in Plasma_Metabolites, joined by ID. |
| Source_FigureS2 | Paired concentration inputs for the opicapone sensitivity cohort (*n* = 14), with substituted 84-h baseline values where available. The worksheet name is retained from the source workbook. |
| Source_Figure3 | Fig. S1 participant values and adjusted changes; fitted-model coefficients, residual degrees of freedom, and conventional covariance matrix. |
| README | Data dictionary, missing-value conventions, contact information, analysis software versions, resampling specifications, and Fig. S1 calculation instructions. |

Supplementary Data 1 preserves the original individual-level observations and existing calculation formulas. Statistical methods and supporting results are provided in this Supplementary material. Analysis code and additional analytical details are available from the corresponding author upon reasonable request.
